# Nkx2.8 promotes chemosensitivity in bladder urothelial carcinoma via transcriptional repression of MDR1

**DOI:** 10.1038/s41419-022-04947-x

**Published:** 2022-05-24

**Authors:** Zhaohui Zhou, Longbin Xiong, Zeshen Wu, Lijuan Jiang, Yonghong Li, Zhiyong Li, Yulu Peng, Kang Ning, Xiangpeng Zou, Zefu Liu, Jun Wang, Zhen Li, Fangjian Zhou, Zhuowei Liu, Zhiling Zhang, Chunping Yu

**Affiliations:** 1 Department of Urology, Sun Yat-Sen University Cancer Center, Guangzhou, PR China; 2 State Key Laboratory of Oncology in Southern China; Collaborative Innovation Center for Cancer Medicine, Guangzhou, PR China; 3 Huazhong University of Science and Technology Union Shenzhen Hospital (Nanshan Hospital), Shenzhen, PR China

**Keywords:** Cancer therapeutic resistance, Bladder cancer

## Abstract

Multidrug resistance gene 1 (MDR1), a key factor contributing to drug insensitivity, has been associated with treatment failure and poor prognoses in various cancers, including bladder urothelial carcinoma (UC). Here we show that positive Nkx2.8 expression was associated with better prognosis of UC patients received chemotherapy. Patients with positive Nkx2.8 expression had promising prognosis from adjuvant chemotherapy. Enforced expression of Nkx2.8 promotes drug sensitivity of UC cells. Mechanistic investigations showed that Nkx2.8 negatively regulated expression of MDR1 by binds directly to the *MDR1* promoter and transcriptionally represses MDR1 expression. P-gp inhibitor reversed chemosensitivity inhibition by Nkx2.8 scilencing. In clinical UC specimens, expression of Nkx2.8 inversely correlated with P-gp expression, and UC patients with Nkx2.8 positivity and low P-gp expression displayed the best prognosis. Our findings uncovered a new mechanism of chemosensitivity in UC cells and proposing Nkx2.8-MDR1 axis as a novel candidate target for therapeutic intervention of UC.

## Introduction

Chemotherapy is widely used in all stages of bladder urothelial carcinoma (UC). In stage Ta/T1 UC, single dose intravesical instillation of chemotherapy after transurethral resection (TUR) can significantly reduce the five-year recurrence rate from 59% to 45% [1]. In locally advanced or metastasis disease, chemotherapy in the neoadjuvant [2, 3], adjuvant [4, 5] or systemic settings [6, 7] have been associated with beneficial outcomes in UC patients. However, not all patients are equally responsive as some may even have no response to chemotherapy or relapse after a period of response. Despite extensive efforts to enhance the efficacy of chemotherapy [8, 9], the survival outcomes are still not satisfying as the underlying mechanisms contributing to drug insensitivity have not been fully determined [10].

Although several mechanisms have been identified to cause drug insensitivity, the predominant cause is believed to be increased efflux of chemotherapeutic drugs to the extracellular milieu via membrane-embedded drug transporters [11, 12]. P-gp, the most studied drug transporter, is encoded by MDR1 and a member of the ATP-binding cassette superfamily. It has been shown to be overexpressed in various types of cancers including acute myeloid leukemia [13], kidney cancer [14], bladder urothelial carcinoma [15], and more. Most studies aimed to improve the chemosensitivity of cancers by inhibiting P-gp [16]. There were also several studies focusing on upstream of MDR1 and identified factors such as Twist and DAB2IP which could regulate P-gp expression in UC [17, 18]. However, the mechanism by which MDR1 is regulated is complicated and more novel regulators should be identified.

The Human Nk2 homeobox 8 (Nkx2.8) belongs to the NK-2 gene family and is known as a transcription factor which usually binds to DNA sequences containing 5′-(C/T)AAG-3′ motifs [19–21]. Studies showed that Nkx2.8 acted as a tumor suppressor in lung cancer, esophageal cancer and liver cancer [22–24]. In one of our previous study, we established that Nkx2.8 inhibited proliferation by upregulating FOXO3a and suppressing the MEK/ERK pathway, and Nkx2.8 negativity was associated with lymph node metastasis and adjuvant chemotherapy efficacy [25]. We also found that Nkx2.8 could inhibit epithelial-mesenchymal transition (EMT) by binding directly to the promoter region of *TWIST1* and transcriptionally repressing Twist1 expression in UC [26].

Here, we found that high expression of Nkx2.8 was associated with better prognosis in UC patients who received chemotherapy both in public database and our clinical cohort. Then we aimed to investigate the role of Nkx2.8 in drug insensitivity and the related regulatory mechanisms.

## Results

### High Nkx2.8 expression was associated with better prognosis in UC patients received chemotherapy

**Fig. 1:**
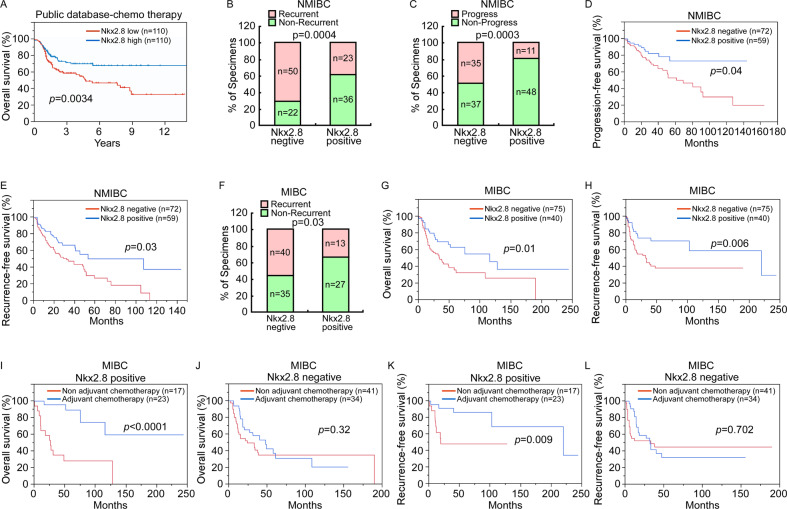
High Nkx2.8 expression associated with better prognosis in UC patients received chemotherapy. **A** Survival analysis of 220 UC patients received chemotherapy from public database by different levels of Nkx2.8. **B** Negative or positive Nkx2.8 expression was relative to the recurrence of NMIBC. p values are indicated (chi-square test). **C** Negative or positive Nkx2.8 expression was relative to the progression of NMIBC. p values are indicated (chi-square test). **D** Comparison of the PFS of NMIBC patients with different levels of Nkx2.8 expression. p values are indicated (log-rank test). **E** Comparison of the RFS of NMIBC patients with different levels of Nkx2.8 expression. p values are indicated (log-rank test). **F** Percentage of MIBC specimens with or without recurrence relative to the negative or positive Nkx2.8 expression. *p* values are indicated (chi-square test). **G** Comparison of the OS of MIBC patients with different levels of Nkx2.8 expression. *p* values are indicated (log-rank test). **H** Comparison of the RFS of MIBC patients with different levels of Nkx2.8 expression. *p* values are indicated (log-rank test). **I** OS curves in MIBC patients in Nkx2.8 positive group who underwent radical cystectomy with or without adjuvant chemotherapy. *p* values are indicated (log-rank test). **J** OS curves in MIBC patients in Nkx2.8 negative group who underwent radical cystectomy with or without adjuvant chemotherapy. *p* values are indicated (log-rank test). **K** RFS curves in MIBC patients in Nkx2.8 positive group who underwent radical cystectomy with or without adjuvant chemotherapy. p values are indicated (log-rank test). **L** RFS curves in MIBC patients in Nkx2.8 negative group who underwent radical cystectomy with or without adjuvant chemotherapy. p values are indicated (log-rank test).

We analyzed a total of 220 UC patients received chemotherapy from public database. Patients were divided into Nkx2.8 high expression and low expression groups according to the median expression of Nkx2.8. The data showed that patients with high expression of Nkx2.8 was associated with better prognosis compared to those with low Nkx2.8 expression (Fig. 1A). To confirm the role of Nkx2.8 in chemotherapy of UC patients, we studied 131 non-muscle invasive bladder cancer (NMIBC) patients who received at least one dose of intravesical instillation of chemotherapy. Immunohistochemical (IHC) assay showed that Nkx2.8 expression was correlated with tumor recurrence (Fig. 1B) and progression of these patients (Fig. 1C). Patients with positive Nkx2.8 had better progression-free survival (PFS) (Fig. 1D) and RFS than patients with negative Nkx2.8 (Fig. 1E). We next retrospectively collected the tumor sample of 115 muscle invasive bladder cancer (MIBC) patients underwent radical cystectomy, in which 57 received adjuvant chemotherapy. The baseline clinicopathologic features of patients with or without adjuvant chemotherapy were shown in Supplementary Table 1. IHC assay also showed that Nkx2.8 expression was correlated with recurrence of these patients (Fig. 1F). Patients with positive Nkx2.8 had better overall survival (OS) (Fig. 1G) and recurrence-free survival (RFS) than those with negative Nkx2.8 (Fig. 1H). More interesting, patients with positive Nkx2.8 expression had promising OS (Fig. 1I) and RFS (Fig. 1K) outcomes from adjuvant chemotherapy, while the OS (Fig. 1J) and RFS (Fig. 1L) were not significantly different between the non-adjuvant chemotherapy group and adjuvant chemotherapy group in patients with negative Nkx2.8.

### Nkx2.8 enhances the chemosensitivity of UC cells in vitro and in vivo

**Fig. 2:**
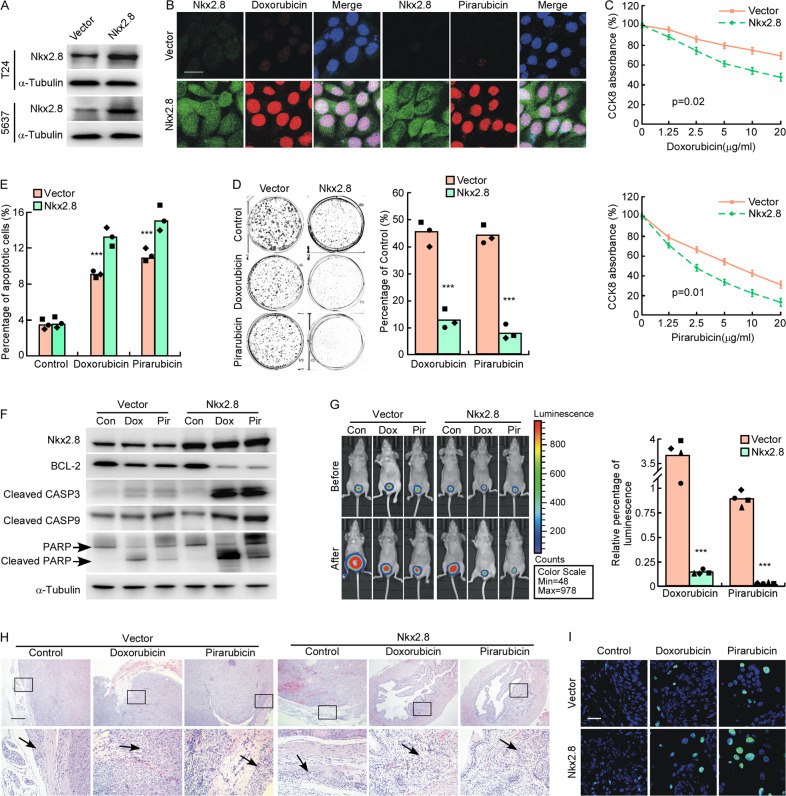
Nkx2.8 enhances the chemosensitivity of UC cells in vitro and in vivo. **A** Western blot analysis of Nkx2.8 in Nkx2.8-overexpressed UC cells. **B** Immunofluorescence of Doxorubicin and Pirarubicin density in T24 with vector or Nkx2.8 overexpressed. Nuclear DNA was stained with DAPI. Scale bar, 20 μm. **C** CCK8 analysis of vector or Nkx2.8 overexpressed T24 after treated by different dose of Doxorubicin (upper) or Pirarubicin (lower) (mean ± SD). *p* values are indicated (two-tailed t test, *n* = 3). **D** Colony formation assays of vector cells or Nkx2.8 overexpressed T24 after treated by Doxorubicin or Pirarubicin (left panel) and quantification (right panel) of colonies (mean ± SD). ***p < 0.0001 (two-tailed *t* test, *n* = 3). **E** The apoptosis rate of vector cells or Nkx2.8 overexpressed T24 after treated by Doxorubicin or Pirarubicin (mean ± SD). ***p < 0.0001 (two-tailed *t* test, *n* = 3). **F** Western blot analysis of apoptotic markers in T24 with vector or Nkx2.8 overexpressed after treated by Doxorubicin or Pirarubicin. **G** Representative images of macroscopic orthophoric bladder urothelial cancer generated from vector or Nkx2.8 overexpressed T24s before or after treated with Doxorubicin (Dox) or Pirarubicin (Pir) (left panel). Quantification of luminescence in orthophoric bladder urothelial cancer (right panel) mean ± SD. ***p < 0.0001 (two-tailed *t* test, *n* = 4). **H** Representative images of H&E staining. Scale bar, 500 μm. **I** TUNEL assay of apoptosis in tumor tissues. Scale bar, 20 μm.

Doxorubicin and Pirarubicin are wildly used in UC chemotherapy. To determine the effect of Nkx2.8 on the drug sensitivity of UC cells, we established Nkx2.8-overexpressed UC cells (Fig. 2A). Doxorubicin (10 μg/mL) and Pirarubicin (5 μg/mL) were used to treat Nkx2.8-overexpressed and vector cells. As Doxorubicin and Pirarubicin have red fluorescence, confocal microscopy was used to detect drug accumulation in UC cells after treatment. Our results showed that drug fluorescence was predominantly found in the nucleus and was increased in Nkx2.8-overexpressed cells compared to vector cells (Figs. 2B and S1A). In cell counting kit-8 (CCK8) assay, the CCK8 absorbance of Nkx2.8-overexpressed cells was found to decrease faster than vector cells as the drug concentration increased (Figs. 2C and S1B). Colony formation assay showed that after treatedwith Doxorubicin or Pirarubicin, Nkx2.8-overexpressed cells formed less and smaller colonies than vector cells, compared to its control cells (Figs. 2D and S1C). Flow cytometry analysis showed that after treated with Doxorubicin or Pirarubicin, compared to its control cells, the apoptotic rate of Nkx2.8-overexpressed cells was much higher than vector cells (Figs. 2E and S1D). We also detected the expression of some apoptotic markers by Western blotting analysis. Our results showed that after treated with Doxorubicin or Pirarubicin, the expression levels of the apoptosis markers, such as cleaved-caspase3, cleaved-caspase9 and cleaved-PARP were upregulated, the expression of BCL-2 was downregulated in Nkx2.8-overexpressed cells (Figs. 2F and S1E). These in vitro data suggested that drug sensitivity of UC cells significantly increased after overexpressed with Nkx2.8.

Next, the in vivo effects of Nkx2.8 on drug sensitivity were evaluated. We constructed orthotopic xenograft UC model by intravesical instilling T24/Nkx2.8 cells or T24/vector cells, which were transfected with luciferase, into nude mice urinary bladder. After 7 days, Doxorubicin (5 mg/kg) or Pirarubicin (5 mg/kg) were instilled into the nude mice urinary bladder intravesically once a week for 4 weeks. Bioluminescent imaging showed that after drug treatment, mice instilled with T24/Nkx2.8 cells had significantly weaker bioluminescent signal than mice instilled with T24/vector cells, compared to control group (Figs. 2G and S1F). Hematoxylin eosin (H&E) staining also showed that after intravesical instilled with drug, mice implanted with T24/Nkx2.8 cells had mild aggressive tumor while those implanted with T24/vector cells had significant muscle invasive tumor (Fig. 2H). The terminal deoxynucleotidyl transferase-mediated dUTP nick end-labeling (TUNEL) staining showed that TUNEL positive cells were much more in T24/Nkx2.8 group than T24/vector group after treated by Doxorubicin or Pirarubicin (Fig. 2I). Briefly, these findings indicated that the overexpression of Nkx2.8 could significantly promote the drug sensitivity of UC cells, both in vitro and in vivo.

### Knocking-down endogenous Nkx2.8 inhibits the in vitro and vivo chemosensitivity of UC cells

**Fig. 3:**
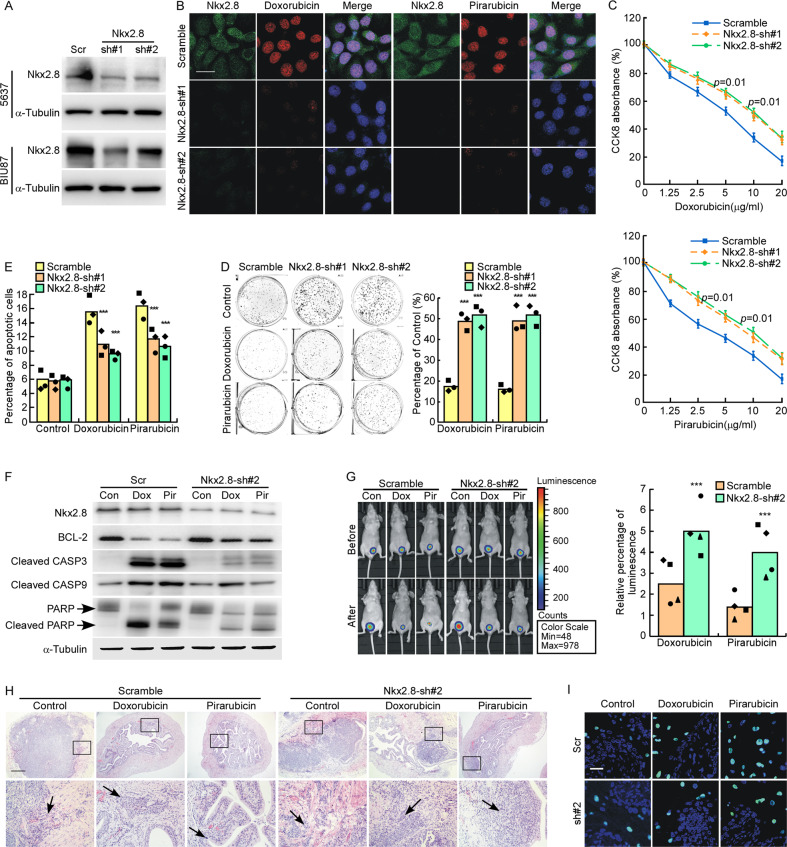
Knocking-down endogenous Nkx2.8 inhibits the in vitro and vivo chemosensitivity of UC cells. **A** Western blot analysis of Nkx2.8 in Nkx2.8-silenced UC cells. **B** Immunofluorescence of Doxorubicin and Pirarubicin density in 5637 with scramble or Nkx2.8 knocking-down. Nuclear DNA was stained with DAPI. Scale bar, 20 μm. **C** CCK8 analysis of scramble or Nkx2.8 knocking-down 5637 after treated by different dose of Doxorubicin (left) or Pirarubicin (right) (mean ± SD). p values are indicated (two-tailed t test, *n* = 3). **D** Colony formation assays of scramble or Nkx2.8 knocking-down 5637 after treated by Doxorubicin or Pirarubicin (left panel) and quantification (right panel) of colonies (mean ± SD). ***p < 0.0001 (twotailed *t* test, *n* = 3). **E** The apoptosis rate of scramble or Nkx2.8 knocking-down 5637 after treated by Doxorubicin or Pirarubicin (mean ± SD). ***p < 0.0001 (two-tailed t test, n = 3). F Western blot analysis of apoptotic markers in scramble or Nkx2.8knocking-down 5637 after treated by Doxorubicin or Pirarubicin. **G** Representative images of macroscopic orthophoric bladder urothelial cancer generated from scramble cells or Nkx2.8 knocking-down 5637 before or after treated with Doxorubicin (Dox) or Pirarubicin (Pir) (left panel). Quantification of luminescence in orthophoric bladder urothelial cancer (right panel) mean ± SD. ***p < 0.0001(two-tailed *t* test, *n* = 4). **H** Representative images of H&E staining. Scale bar, 500 μm. **I** TUNEL assay of apoptosis in tumor tissues. Scale bar, 20 μm.

To further investigate the effect of Nkx2.8 on the drug sensitivity of UC cells, we established Nkx2.8- silenced UC cells (Fig. 3A) and Doxorubicin and Pirarubicin was used to treat Nkx2.8-silenced cells. Our results showed that drug fluorescence was decreased in Nkx2.8-silenced cells compared to scrambled cells (Figs. 3B and S2A). In CCK8 assay, the CCK8 absorbance of Nkx2.8-silenced cells decreased much slower than scrambled cells as the drug concentration increased (Figs. 3C and S2B). Colony formation assay showed that after treated with Doxorubicin or Pirarubicin, Nkx2.8-silenced cells formed greater number and larger colonies than scrambled cells, compared to control cells (Figs. 3D and S2C). Flow cytometry data showed that after treated with Doxorubicin or Pirarubicin, the apoptotic rate of Nkx2.8-silenced cells was much lower than scrambled cells, compared to control cells (Figs. 3E and S2D). Western blotting analysis showed that after treated with Doxorubicin or Pirarubicin, the expression levels of cleaved-caspase3, cleaved-caspase9 and cleaved-PARP were downregulated, the expression of BCL-2 was upregulated in Nkx2.8-silenced cells (Figs. 3F and S2E). These findings suggested that the in vitro drug sensitivity was significantly decreased in Nkx2.8-silenced cells.

We then constructed an orthotopic xenograft urinary bladder cancer model by intravesical instilling 5637/Nkx2.8-shRNA cells or 5637/scrambled cells, which were transfected with luciferase, into the nude mice urinary bladder. After 7 days, Doxorubicin or Pirarubicin was instilled into the nude mice’s urinary bladder intravesically once a week for 4 weeks. Bioluminescent imaging showed that after drug treatment, mice instilled with 5637/Nkx2.8-shRNA cells had significantly stronger bioluminescent signal than mice instilled with 5637/scrambled cells, compared to control group (Figs. 3G and S2F). H&E staining also showed that after intravesical instilled with drug, mice implanted with 5637/Nkx2.8-shRNA cells showed significant muscle invasive tumor while mice implanted with 5637/scramble cells had mild aggressive tumor (Fig. 3H). TUNEL staining showed that TUNEL positive cells were much less in 5637/Nkx2.8-shRNA group than 5637/scramble group after treated by Doxorubicin or Pirarubicin (Fig. 3I). These data confirmed that the knock-down of endogenous Nkx2.8 inhibited the chemosensitivity in UC cells.

### Nkx2.8 downregulated the expression of MDR1 in UC cells

**Fig. 4:**
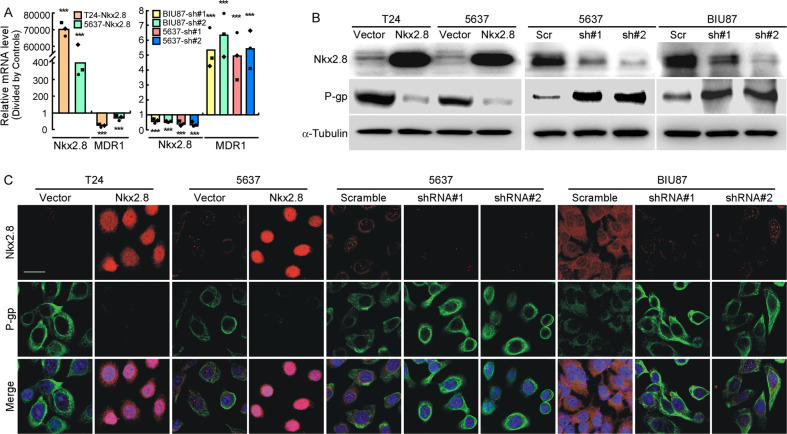
Nkx2.8 represses MDR1 expression in UC cells. **A** Real-time PCR analysis of Nkx2.8 and MDR1 mRNA expression in T24 and 5637 with vector or Nkx2.8 overexpressed (left panel) or 5637 and BIU87 with scramble or Nkx2.8 knocking-down (right panel). Transcript levels were normalized to GAPDH (mean ± SD). ***p < 0.0001 (two-tailed *t* test, *n* = 3). **B** Western blot analysis of Nkx2.8 and P-gp expression in the indicated cells, α-Tubulin was used as a loading control. **C** Immunofluorescence analysis of Nkx2.8 and P-gp expression in indicated cells. The blue signal signifies nuclear DNA staining with DAPI. Scale bar, 20 μm.

To explore the underlying mechanism of Nkx2.8 promoting drug sensitivity, we detected the relationship between Nkx2.8 and several drug transporters, including MDR1, MRP1, MRP7, and BCRP, using quantitative PCR. The results showed that Nkx2.8 down-regulated MDR1 mRNA expression but had no significant effect on the other above-mentioned gene mRNA expressions (Fig. S3). We further investigated the relationship between Nkx2.8 and MDR1 in Nkx2.8-overexpressed and Nkx2.8-silenced cells. Quantitative PCR analysis showed that MDR1 mRNA was significantly decreased in cells overexpressing exogenous Nkx2.8 compared to vector cells, while MDR1 mRNA was markedly up-regulated in Nkx2.8-silenced cells, compared to scrambled cells (Fig. 4A). Western blotting analysis (Fig. 4B) and confocal microscopy assay (Fig. 4C) also showed that P-gp expression was significantly decreased in Nkx2.8-overexpressed cells, compared to vector cells, and was markedly up-regulated in Nkx2.8-silenced cells compared to scrambled cells. These results indicated that Nkx2.8 inhibited MDR1 expression at both mRNA and protein levels in UC cells.

Studies showed that several factors such as Twist could regulate P-gp expression in UC [17, 18]. For the reason that we have demonstrated that Nkx2.8 transcriptionally represses Twist1. So it is possible that Nkx2.8 represses P-gp may be caused by the downregulated Twist1. To verify this, we silenced Twist1 expression with *TWIST1* siRNA in Nkx2.8-silenced cells and detected P-gp expression by Western blot. The result showed that silenced Twist1 barely impact the expression of P-gp in Nkx2.8-silenced cells (Fig. S4). This data suggested that Nkx2.8 represses P-gp is independent of Twist1.

### Nkx2.8 binds to the *MDR1* promoter locus and transcriptionally represses *MDR1*

**Fig. 5:**
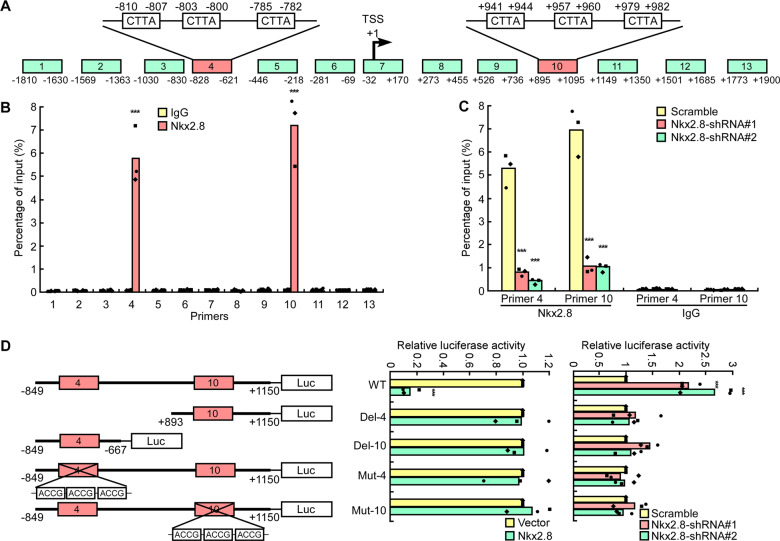
Nkx2.8 binds to the *MDR1* promoter locus and transcriptionally represses *MDR1*. **A** Schematic representation of the promoter region of *MDR1*. Precipitated DNA was amplified in a PCR assay using primers specific for regions 1-13. TSS: Transcriptional start site. **B** ChIP analysis was performed using an anti-Nkx2.8 antibody or anti-IgG antibody to identify Nkx2.8 binding sites on the *MDR1* promoter in 5637 cells (mean ± SD). **C** ChIP analysis of Nkx2.8 binding efficiency in 5637 cells expressing the scrambled shRNA or Nkx2.8 shRNA (mean ± SD). **D** Transactivities of Nkx2.8 on serial *MDR1* promoter fragments as indicated in 5637 cells (mean ± SD). **p < 0.001 (two-tailed *t* test, *n* = 3).

We then tried to explore the mechanism by which Nkx2.8 inhibited MDR1 expression. As Nkx2.8 is known as a transcriptional factor that binds to special DNA sequences in promoters [20, 21]. As we showed in our previous study [26], Nkx2.8 could bind directly to the *TWIST1* promoter at both loci which extend from -1510 bp to -1472 bp and from +774 bp to + 801 bp. Here, we investigated whether Nkx2.8 could act as a transcriptional factor for *MDR1*. Interestingly, we also identified two potential binding sites of Nkx2.8 in the *MDR1* promoter, each includes three adjacent core sequences (Fig. 5A). Therefore, we speculated that Nkx2.8 could bind to the promoter locus and regulate *MDR1* transcription. ChIP assay was used to verify this speculation. We detected 13 *MDR1* promoter loci. As expected, Nkx2.8 bound to the 4th and 10th loci of the *MDR1* promoter. These loci extend from −810 bp to −782 bp and from +941 bp to + 982 bp, respectively (Figs. 5A, B). The binding was suppressed when Nkx2.8 was silenced (Fig. 5C), confirming that Nkx2.8 could directly target the *MDR1* promoter. Luciferase reporter assay also showed that overexpressed Nkx2.8 decreased the luciferase activity driven by the wild type *MDR1* promoter while silencing Nkx2.8 increased the luciferase activity driven by the *MDR1* promoter. To confirm this function of Nkx2.8, we constructed mutated *MDR1* promoters at the 4th or 10th loci. Our findings showed that neither the overexpression nor knockdown of Nkx2.8 had effect on the luciferase activity levels of *MDR1* promoters containing a deleted or mutated 4th or 10th locus (Fig. 5D), indicating that both the 4th and the 10th locus were required for Nkx2.8 to regulate the *MDR1* promoter.

### Inhibition of P-gp restores the decreased drug sensitivity induced by Nkx2.8-silencing

**Fig. 6:**
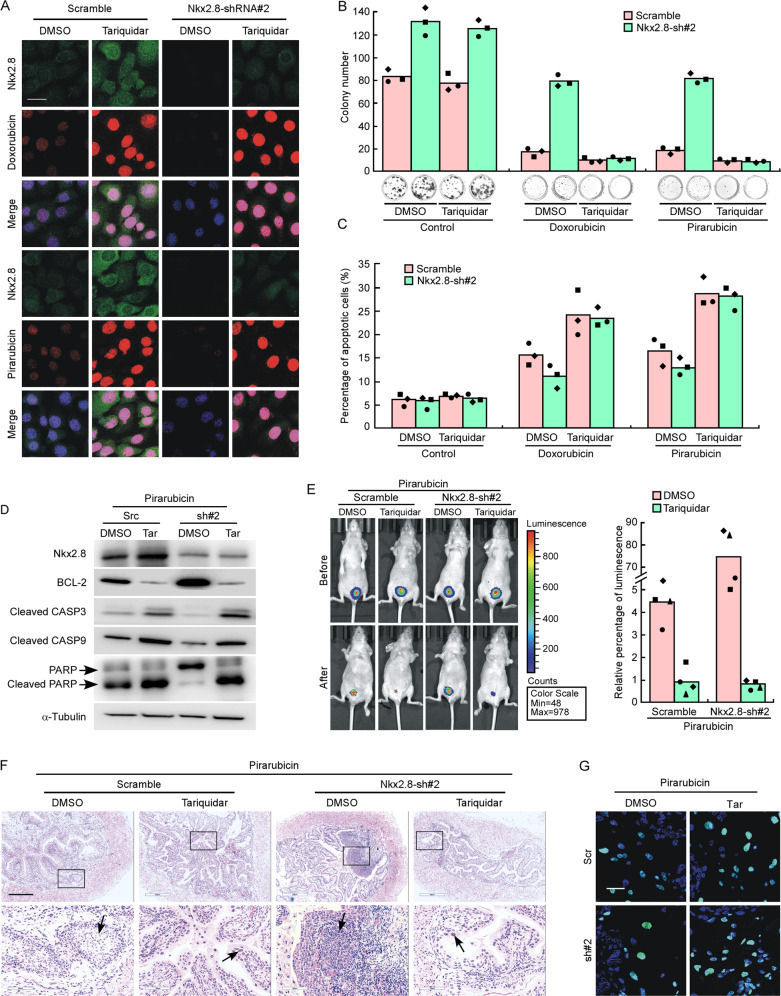
Inhibition of P-gp restores the decreased drug sensitivity induced by Nkx2.8-silencing. **A** Immunofluorescence analysis of Doxorubicin or Pirarubicin density in scramble or Nkx2.8 knocking-down 5637 treated with DMSO or P-gp inhibitor Tariquidar. The blue signal signifies nuclear DNA staining with DAPI. Scale bar, 20 μm. **B** Colony formation assay of scramble or Nkx2.8 knocking-down 5637 after treated by Doxorubicin or Pirarubicin and adding with DMSO or Tariquidar (mean ± SD). **C** The apoptosis rate of scramble or Nkx2.8 knocking-down 5637 after treated by Doxorubicin or Pirarubicin and adding with DMSO or Tariquidar (mean ± SD). **D** Western blot analysis of apoptotic markers in Nkx2.8-silenced UC cells after treated by Pirarubicin and adding with DMSO or Tariquidar. **E** Representative images of macroscopic orthophoric bladder urothelial cancer generated from scramble or Nkx2.8 knocking-down 5637 before or after treated with Pirarubicin and adding DMSO or Tariquidar (left panel). Quantification of luminescence in orthophoric bladder urothelial cancer (right panel) mean ± SD. **F** Representative images of H&E staining. Scale bar, 500 μm. **G** TUNEL assay of apoptosis in tumor tissues. Scale bar, 20 μm.

Here, the functional correlation between MDR1 and Nkx2.8 was further investigated. To test whether the ablation of MDR1 expression could restore the decrease drug sensitivity induced by Nkx2.8-silencing, we used P-gp inhibitor Tariquidar to treat Nkx2.8-silenced cells. Confocal microscopy showed that after treatment with Tariquidar (6.5 μM), drug fluorescence was significantly stronger in both Nkx2.8-silenced cells and scramble cells compared to DMSO treated cells, indicating that P-gp inhibitor could recover the decreasing of drugs in cells by Nkx2.8 silencing (Figs. 6A and S5A). Colony formation assay showed that Tariquidar recovered the enhanced colony formation ability of Nkx2.8-silenced cells after treatment with Doxorubicin or Pirarubicin (Figs. 6B and S5B). Flow cytometry data also showed that Tariquidar recovered the decreased apoptotic rate of Nkx2.8-silenced cells after treatment with Doxorubicin or Pirarubicin (Figs. 6C and S5C). Western blotting analysis showed that after treated with Doxorubicin or Pirarubicin, the expression levels of cleaved-caspase3, cleaved-caspase9, cleaved-PARP and BCL-2 were recovered by Tariquidar (Figs. 6D and S5D).

In vivo experiment also showed that Tariquidar could restore the decrease of chemosensitivity induced by Nkx2.8-silencing. Figs. 6E and S5E showed that after treatment with Pirarubicin, the tumor sizes of mice bearing either 5637/Nkx2.8-shRNA or scramble cells were much smaller when treated with Tariquidar, compared to those treated with DMSO. H&E staining also showed that after intravesical instilled with drug, mice implanted with either 5637/Nkx2.8-shRNA or scramble cells showed mild aggressive tumor when treated with Tariquidar (Fig. 6F). TUNEL staining showed that after treated with Pirarubicin, the number of TUNEL positive cells were almost the same in 5637/Nkx2.8-shRNA group and 5637/scramble group after treated with Tariquidar (Fig. 6G). These findings suggested that inhibition of P-gp restored the decrease of drug sensitivity induced by Nkx2.8-silencing both in vitro and vivo.

### Survival analyses revealed the optimal prognosis prediction of Nkx2.8 ratio

To confirm the above findings, the expression levels of P-gp were also detected in 131 NMIBC and 115 MIBC tissue specimens via immunohistochemical (IHC) assay. In NMIBC patients, P-gp expression was also correlated with recurrence and progression (Fig S6A, B). Patients with high P-gp expression had worse RFS (Fig. S6C) and PFS (Fig. S6D) than patients with low P-gp expression. In MIBC patients, P-gp was also correlated with recurrence (Fig. S6E). High P-gp expression patients had worse OS (Fig. S6F) and RFS (Fig. S6G) than low P-gp expression patients. However, patients had promising OS outcomes from adjuvant chemotherapy in both low P-gp expression group (Fig. S6H) and high P-gp expression group (Fig. S6I). So, P-gp plays a negative role in chemotherapy effect in UC patients.

**Fig. 7:**
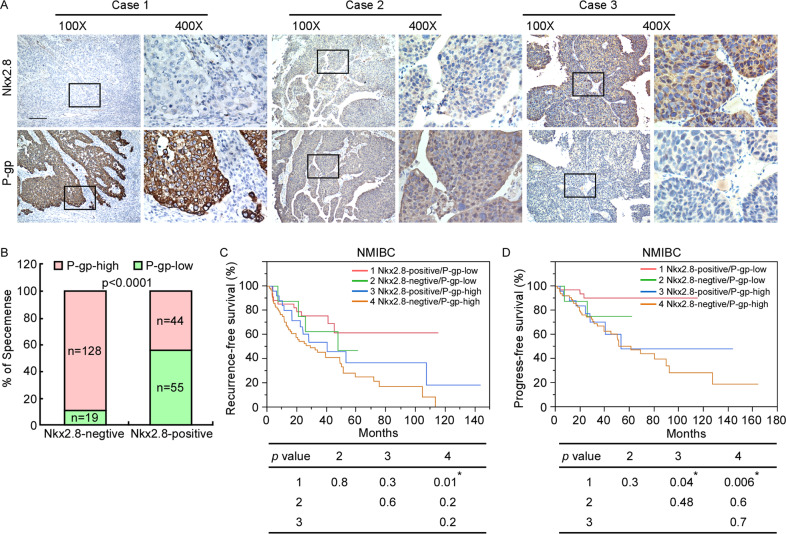
Clinical relevance of Nkx2.8 and Twist1 in human UC. **A** Nkx2.8 levels were negatively associated with P-gp expression in 246 human UC specimens. Three representative cases are shown (scale bar, 200 μm). **B** Percentage of UC specimens showing negative or positive Nkx2.8 expression relative to the level of P-gp. p values are indicated (chi-square test). **C** Comparison of the RFS times of NMIBC patients with different levels of Nkx2.8 and P-gp expression combination. p values are indicated (log-rank test). **D** Comparison of the PFS times of NMIBC patients with different levels of Nkx2.8 and P-gp expression combination. p values are indicated (log-rank test).

As shown in Figs. 7A, B, Nkx2.8 expression was inversely correlated with P-gp expression. In 131 NMIBC patients, Nkx2.8 and P-gp were both associated with the recurrence and progression (Supplementary Table 2). In addition, multivariate survival analyses indicated that the Nkx2.8 and P-gp expression were independent prognostic factors for RFS and PFS (Supplementary Table 3 and 4). Patients with negative Nkx2.8 and high P-gp had worse RFS (Fig. 7C) and PFS (Fig. 7D). In summary, our results reveal that an inverse relationship exists between Nkx2.8 and P-gp expression and that positive Nkx2.8 and low P-gp expression lead to better outcomes in UC patients than negative Nkx2.8 or high P-gp expression.

## Discussion

Chemotherapy failure is the main reason for the relapse of various tumors, including UC. Hence, a better understanding of the molecular mechanism of UC chemotherapeutical resistance has significant clinical relevance. Our previous work of Nkx2.8 in UC has demonstrated that Nkx2.8 could inhibit UC cell proliferation [25] and EMT [25]. In patients with adjuvant chemotherapy, those with negative Nkx2.8 expression had a much higher risk of recurrence than patients with positive Nkx2.8 [25]. To clarify the relationship between Nkx2.8 and chemotherapeutic effect, we investigated the role of Nkx2.8 in the chemosensitivity of UC cells. As clearly showed in the results, Nkx2.8 up-regulated UC cells were more sensitive to Doxorubicin and Pirarubicin while UC cells with down-regulated Nkx2.8 were insensitive to chemotherapy. Those results demonstrated a novel function of Nkx2.8, which is characteristic of enhancing the chemosensitivity of UC cells.

A great number of researchers attributed chemotherapeutical resistance to MDR1 and its encoded protein P-gp [12]. In UC, MDR1 mRNA and P-gp were found highly expressed in high grade, residual and recurrent tumors after chemotherapy, and could also predict poor outcomes after neoadjuvant chemotherapy [15, 27]. Several factors such as Twist and DAB2IP are reported to regulate P-gp expression in UC [17, 18]. In this current study, we found that Nkx2.8 could directly bind to *MDR1* promoter and transcriptionally repress MDR1 expression. Our study has demonstrated a novel mechanism for regulating MDR1 which may provide a new target to promote chemosensitivity in UC.

More specifically, the promoter region of *MDR1* contained two potential binding sites for Nkx2.8 located on either side of the transcription start site, each containing three adjacent core sequences. Mutation of any region led to the failure of repression by Nkx2.8, showing that Nkx2.8 should bind to both loci to inhibit the promoter of *MDR1*. This finding is similar to the binding way of Nkx2.8 to *TWIST1* promoter, which also contain two binding areas and are both required [26]. Our findings further illustrated that this maybe a specified pattern of Nkx2.8 to regulate downstream factors which can be used to identify new targets of Nkx2.8. To explore the mechanisms of how Nkx2.8 inhibits transcriptional activity of MDR1, we detected the methylation level of MDR1 promoter in Nkx2.8-overexpressed UC cells and Nkx2.8-silenced UC cells as promoter methylation always inhibits gene expression. But unfortunately we only got a negative result. So the mechanisms need further exploration. In summary, this study identified a new upstream molecule of MDR1 and identified a novel mechanism how Nkx2.8 regulates MDR1.

In clinical setting, the expression of Nkx2.8 and P-gp in UC tissue demonstrated significant association with clinicopathologic parameters. Our results showed that positive expression of Nkx2.8 was associated with lower recurrence rate and progression rate. Also, Nkx2.8 negativity showed discrimination on the effectiveness of adjuvant chemotherapy in MIBC. Conversely, high expression of P-gp indicated a higher recurrence rate and progression rate. Importantly, the expression of Nkx2.8 and P-gp was negatively associated. This is accordant with our in vivo and vitro results. This inverse relationship between Nkx2.8 and P-gp expression has an impact on the prognoses of UC patients, as patients displaying Nkx2.8 positivity and low P-gp expression have a better prognosis than patients displaying Nkx2.8 negativity or high P-gp expression. Although robust evidence showing that Nkx2.8 could directly inhibit the transcription of MDR1, not every patient with positive Nkx2.8 expression had a low expression of P-gp. Our results showed that 44 of 99 patients with positive Nkx2.8 expression exhibited high expression of P-gp, suggesting that there are probably other unrecognized mechanisms affecting the inhibition of Nkx2.8 on P-gp. 19 of 147 cases with negative Nkx2.8 expression showed low P-gp expression, implying that P-gp inhibition in these patients occurs independently of Nkx2.8 expression. Thus, additional studies are needed to identify other upstream regulators of P-gp.

In conclusion, our findings serveed an extensive explanation of the mechanism underlying the chemosensitivity in UC, as we elucidated the crucial role of the Nkx2.8-MDR1 pathway in drugsensitivity of UC and may have thus identified novel therapeutic targets for the treatment of UC.

## Materials and methods

### Cell lines

The bladder cancer cell lines T24 and 5637 were obtained from the American Tissue Culture Collection (ATCC, Rockville, MD). The BIU87 was obtained from the Institute of Urology at the First Affiliated Hospital of Peking University as a gift. All cell lines were maintained in RPMI 1640 (Invitrogen) supplemented with 10% FBS (HyClone), penicillin (100 units/mL), and streptomycin (100 units/mL) and tested to ensure mycoplasma free. All cell lines used in this study were authenticated 3 months before the beginning of the study based on viability, recovery, growth, morphology, and isoenzymology by the supplier, and all the cell lines have not been in culture for more than 2 months.

### Plasmids and retroviral infection

The wild-type human *MDR1* promoter and the *MDR1* promoter with a deletion or mutation of the Nkx2.8 binding sites were individually cloned into the pGL3 luciferase reporter plasmid (Promega). UC cells with endogenous silencing of Nkx2.8 and cells with the forced expression of exogenous Nkx2.8 were generated as previously described [26]. The T24 cells exhibited low expression of Nkx2.8 and were infected with retroviruses carrying pBabe-Nkx2.8. The BIU87 cells showed high expression levels of Nkx2.8 and were infected with retroviruses carrying pSuper-retro-Nkx2.8-shRNAs. The 5637 cells showed moderate expression levels of Nkx2.8 and thus were infected with retroviruses carrying either pBabe-Nkx2.8 or pSuper-retro-Nkx2.8-shRNAs. Stable cell lines were selected with 0.5 g/ml puromycin for 10 days after transfection. The pBabe-Nkx2.8 and pSuper-retro-Nkx2.8 RNAi(s) were generated as described previously [26]. Cell lysates, which were prepared from pooled populations of cells in sample buffer, were fractionated by sodium dodecyl sulfate-polyacrylamide gel electrophoresis to confirm Nkx2.8 protein levels.

### RNA extraction, quantitative real-time PCR

Total RNA samples from cultured cells were extracted using Trizol reagent (Invitrogen) according to the manufacturer’s instructions. The sequences of the primers are listed in Supplemental Table 5. Quantitative real-time PCR (qRT-PCR) was performed using an ABI PRISM 7500 Sequence Detection System (Applied Biosystems). For the analysis of qRT-PCR data, the expression of each product were calculated by the Relative Quantification (ΔΔCT) method and normalized to housekeeping gene glyceraldehyde-3 phosphate dehydrogenase (GAPDH), and further presented as “Relative mRNA level” compared to relative controls (Vector or Scr cells), which were defined as “1”.

### Western blot and immunofluorescence analyses

Western blot and immunofluorescence analyses were performed according to standard methods as described previously using anti-Nkx2.8 (Santa Cruz Boitechnology, Inc), anti-P-gp (Abcam), anti-PARP (Cell Signaling Technology), anti-caspase-3 (Cell Signaling Technology), anti-caspase-7 (Cell Signaling Technology), anti-caspase-9 (Cell Signaling Technology) and anti-Bcl2 (Cell Signaling Technology) antibodies. For the Western blot assays, anti-α-Tubulin antibody (Sigma) was used as a loading control. For immunofluorescence analysis, the coverslips were counterstained with 4′, 6-diamidino-2-phenylindole and imaged with a confocal laser-scanning microscope (Olympus FV1000).

### Patient information and immunohistochemistry

From the public database, RNA sequences and corresponding clinical data of 220 UC patients were directly downloaded from the series of GSE13507 (*n* = 27), GSE31684 (*n* = 35), and GSE70691 (*n* = 49) in the GEO database and obtained from the TCGA database (*n* = 109) using the TCGA bio links package R software (version 4.0.3).

The study has been approved by Institutional Review Board of Sun Yat-sen University Cancer Center, and the study was performed in accordance with Declaration of Helsinki. Written informed consent was obtained from the patients before the study began. Nkx2.8 and P-gp expression in the paraffin-embedded UC tissues of 131 NMIBC and 115 MIBC were detected by immunohistochemistry (IHC) using an anti-Nkx2.8 and anti-P-gp antibody. 131 NMIBC patient information was summarized in supplementary Table 1. All the 131 cases of NMIBC received at least one dose of intravesical instillation of chemotherapy. Another 115 cases of MIBC who underwent radical cystectomy were also included in this immunohistochemistry analysis and 57 of them received adjuvant chemotherapy(3 cycles of gemcitabine plus cisplatinum). The baseline clinicopathologic features of these patients were listed in the Supplemental Table 1. The method for scoring Nkx2.8 has been previously reported [26]. For the P-gp labeling index, we used the following scoring system. In brief, the proportion of positive cells in the stained sections was evaluated at ×200 magnification, and the mean value of 10 representative fields analyzed from each section was recorded. The proportion of positive cells was scored as follows: <25%, 1; 25% to 50%, 2; 50% to 75%, 3; and >75%, 4. We judged the intensity of P-gp staining according to 4 categories: negative, 0; weak, 1; moderate, 2; and strong, 3. We used the product of the staining intensity score and the proportion of positive tumor cells as the staining index. Then, the scores were divided into 2 groups (0–4, low expression; 5–12, high expression).

### Reagents

Pirarubicin and Doxorubicin and Tariquidar were obtained from Selleck, CCK8 reagents from DOJINDO.

### Cell counting kit-8 (CCK8) assay

Cells were seeded into 96-well plates at 2000 cells/well and allowed to attach overnight. Dimethylsulfoxide (DMSO; Merck Millipore, Darmstadt, Germany), Doxorubicin or Pirarubicin was added to the medium at different concentrations for the following days. After 48 h, CCK8 reagents were added to each well and then incubated at 37 °C for 2 h. Absorbance at 450 nm were read using an automated plate reader.

### Apoptosis assays

To analyze cell apoptosis levels, cells were seeded into 6-well plates at 1 × 10^5^ cells/well. After overnight attachment, DMSO, Doxorubicin or Pirarubicin was added to treat the cells for 48 h. Then, floating and attached cells were collected and stained with Annexin V-APC (allophycocyanin; KeyGEN, Jiangsu, China) and DAPI (4′ 6-diamidino-2-phenylindole; KeyGEN, Jiangsu, China). Apoptosis rates were measured by flow cytometry and analyzed by FlowJo software.

### TUNEL staining

TUNEL staining was performed using the TUNEL cell apoptosis detection kit (Beyotime, China) according to the manufacturer’s instructions. Briefly, sections (4 µm) were deparaffinized in xylene, rehydrated in decreasing concentrations of ethanol, boiled for 10 min, and digested in 0.5% pepsin for 60 min at 37 °C, before endogenous peroxidase was blocked in 3% hydrogen peroxide. Then put TUNEL for 1 h at 37 °C. The FITC-labeled TUNEL-positive cells were imaged under a fluorescent microscope by using 488 nm excitation and 530 nm emission. The cells with green fluorescence were defined as apoptotic cells.

### Colony formation assay

Cells were plated on 6-well plates (0.5 × 10^3^ cells per plate). After overnight attachment, DMSO, Doxorubicin or Pirarubicin was added to treat the cells. After 10 days, the colonies were stained with 1% crystal violet for 30 s after fixation with 10% formaldehyde for 5 min.

### Chromatin immunoprecipitation (ChIP) assays

The ChIP assay was performed according to the protocol described previously [26]. In brief, cells were plated at a concentration of 2×10^6^ cells per 100-mm diameter dish and cross-linked with 1% formaldehyde for 10 min. The cells were trypsinized and resuspended in lysis buffer. The nuclei were then isolated and sonicated to shear the DNA into 500-bp to 1-kb fragments, which was verified by agarose gel electrophoresis. Equal aliquots of chromatin supernatants were incubated with 1 μg of anti-Nkx2.8 antibody (Santa Cruz Biotechnology, Inc.) or an anti-IgG antibody (Sigma) overnight at 4°C with rotation. DNA was extracted, and the *MDR1* promoter was amplified with a quantitative PCR assay. All the ChIP assays were performed three times, and representative results were presented. All the sequences of the PCR primers are shown in Supplemental Table 6.

### Luciferase assay

Twenty thousand cells were seeded in triplicate in 48-well plates and allowed to settle for 24 h. Using the Lipofectamine 2000 reagent according to the manufacturer’s recommendations, 100 ng of luciferase reporter plasmids containing different fragments of the *MDR1* promoter, or the control luciferase plasmid, plus 1 ng of pRL-TK Renilla plasmid (Promega) was transfected into the cells. The luciferase and Renilla signals were measured 24 h after transfection using the Dual-Luciferase Reporter Assay Kit (Promega) according to a protocol provided by the manufacturer.

### Xenografted tumor model and hematoxylin and eosin (H&E) staining

BALB/c-nu mice (4–5 weeks of age, female, 18–20 g) were purchased from Charles River Laboratories. All the experimental procedures were approved by the Institutional Animal Care and Use Committee of Sun Yat-sen University. For the orthotopic xenograft bladder cancer model, the urothelial bladder was washed with PBS and then scratched with the catheter tip before instilling 100 mL of 2 × 10^6^ cells through a small catheter. The urethra was temporarily closed with a single, sterile suture at the distal part of the urethra, thus retaining the cells in the urothelial bladder for 2 h. The BALB/c nude mice were randomly divided into 4 groups (*n* = 6/group). Each group of mice was instilled with T24/vector cells, T24/Nkx2.8 cells, 5637/scrambled cells, and 5637/Nkx2.8 RNAi cells transfected with luciferase. Xenograft implantation was confirmed by the presence of bioluminescence activity 1 week after cell implantation. Pirarubicin (5 mg/kg), Doxorubicin (5 mg/kg), and negative control (saline) were instilled into the urothelial bladder through a small catheter and kept for 1 h. Then bioluminescence activity was detected every 2 weeks. For bioluminescent evaluation, each mouse received 150 mg luciferin/kg body weight through intraperitoneal injection. Imaging of the mice was then conducted in anesthetized conditions with the IVIS Lumins III. After 8 weeks, the bioluminescence activity was detected again, the animals were sacrificed, and the urothelial bladder was excised, weighed, and embedded in paraffin. Serial 6.0-mm sections were cut and subjected to hematoxylin and eosin (H&E) staining with Mayer’s hematoxylin solution. Images were captured using the AxioVision Rel.4.6 Computerized Image Analysis System (Nikon Eclipse 80i).

### Statistical analysis

Statistical tests for the data analysis included Fisher’s exact test, the log-rank test, and Student’s two-tailed *t*-test. The data were presented as the means ± SD. All analysis were performed in R version 4.0.3 (http://www.r-project.org/) and a value of *P* < 0.05 was considered statistically significant.

## Supplementary information

Supplemental Material

Supplemental Figure 1

Supplemental Figure 2

Supplemental Figure 3

Supplemental Figure 4

Supplemental Figure 5

Supplemental Figure 6

Original Data File

Reproducibility checklist

## Data Availability

All data needed to evaluate the conclusions in the paper are present in this published article and its supplementary information files. Additional data related to this paper may be requested from the corresponding author on reasonable request.
