# Supplementary material for: Nkx2.8 promotes chemosensitivity in bladder urothelial carcinoma via transcriptional repression of MDR1
Source: Cell Death Dis. 2022 May 24;13(5):492. doi: 10.1038/s41419-022-04947-x (PMC9130207; doi:10.1038/s41419-022-04947-x)
Supplement: Supplementary file 1 — Supplemental Material [file 41419_2022_4947_MOESM1_ESM.doc]

Supplemental Data

**Supplemental figure legends**

**Figure S1. Nkx2.8 enhances the chemosensitivity of UC cells *in* *vitro* and *in vivo*.**

**(A)** Immunofluorescence of Doxorubicin and Pirarubicin density in 5637 with vector or Nkx2.8 overexpressexd. Nuclear DNA was stained with DAPI. Scale bar, 20 μm.

**(B)** CCK8 analysis of vector or Nkx2.8 overexpressexd 5637 after treated by different does of Doxorubicin (left) or Pirarubicin (right) (mean ± SD). p values are indicated (two-tailed t test, n = 3).

**(C)** Colony formation assays of vector cells or Nkx2.8 overexpressexd 5637 after treated by Doxorubicin or Pirarubicin (left panel) and quantification (right panel) of colonies (mean ± SD). ***, p<0.0001 (two-tailed t test, n = 3).

**(D)** The apoptosis rate of vector cells or Nkx2.8 overexpressexd 5637 after treated by Doxorubicin or Pirarubicin (mean ± SD). ***, p<0.0001 (two-tailed t test, n = 3).

**(E)** Western blot analysis of apoptotic markers in 5637 with vector or Nkx2.8 overexpressed after treated by Doxorubicin or Pirarubicin.

**(F)** Time sequential of luminescence in orthophoric bladder urothelial cancer generated from vector or Nkx2.8 overexpressed T24s before or after treated with Doxorubicin or Pirarubicin.

**Figure S2. Knocking-down endogenous Nkx2.8 inhibits the *in vitro* and *vivo* chemosensitivity of UC cells.**

**(A)** Immunofluorescence of Doxorubicin and Pirarubicin density in BIU87 with scramble or Nkx2.8 knocking-down. Nuclear DNA was stained with DAPI. Scale bar, 20 μm.

**(B)** CCK8 analysis of scramble or Nkx2.8 knocking-down BIU87 after treated by different does of Doxorubicin (left) or Pirarubicin (right) (mean ± SD). p values are indicated (two-tailed t test, n = 3).

**(C)** Colony formation assays of scramble or Nkx2.8 knocking-down BIU87 after treated by Doxorubicin or Pirarubicin (left panel) and quantification (right panel) of colonies (mean ± SD). ***, p<0.0001 (two-tailed t test, n = 3).

**(D)** The apoptosis rate of scramble or Nkx2.8 knocking-down BIU87 after treated by Doxorubicin or Pirarubicin (mean ± SD). ***, p<0.0001 (two-tailed t test, n = 3).

**(E)** Western blot analysis of apoptotic markers in scramble or Nkx2.8 knocking-down BIU87 after treated by Doxorubicin or Pirarubicin.

**(F)** Time sequential of luminescence in orthophoric bladder urothelial cancer generated from scramble cells or Nkx2.8 knocking-down 5637 before or after treated with Doxorubicin or Pirarubicin.

**Figure S3. Relationship between Nkx2.8 and MDR1, MRP1, MRP7 and BCRP mRNA expression.**

**(A)** Real-time PCR analysis of Nkx2.8 and MDR1, MRP1, MRP7, and BCRP mRNA expression in T24 and 5637 with vector or Nkx2.8 overexpressed.

**(B)** Real-time PCR analysis of Nkx2.8 and MDR1, MRP1, MRP7, and BCRP mRNA expression in 5637 and BIU87 with scramble or Nkx2.8 knocking-down.

**Figure S4.** **Western blot** **analysis of** **P-gp expression** **in Nkx2.8-silenced cells with Src or *TWIST1* siRNA.**

**Figure S5. Inhibition of P-gp restores the decreased drug sensitivity induced by Nkx2.8-silencing.**

**(A)** Immunofluorescence analysis of Doxorubicin or Pirarubicin density in scramble or Nkx2.8 knocking-down BIU87 treated with DMSO or P-gp inhibitor Tariquidar. The blue signal signifies nuclear DNA staining with DAPI. Scale bar, 20 μm.

**(B)** Colony formation assay of scramble or Nkx2.8 knocking-down BIU87 after treated by Doxorubicin or Pirarubicin and adding with DMSO or Tariquidar (mean ± SD).

**(C)** The apoptosis rate of scramble or Nkx2.8 knocking-down BIU87 after treated by Doxorubicin or Pirarubicin and adding with DMSO or Tariquidar (mean ± SD).

**(D)** Western blot analysis of apoptotic markers in Nkx2.8-silenced BIU87 cells after treated by Pirarubicin and adding with DMSO or Tariquidar.

**(E)** Time sequential of luminescence in orthophoric bladder urothelial cancer generated from Scramble or Nkx2.8-silenced BIU87s before or after treated by Pirarubicin and adding with DMSO or Tariquidar.

**Figure S6. High P-gp expression correlated with worse prognosis in UC patients received chemotherapy**

**(A)** Different levels of P-gp expression was relative to the recurrence of NMIBC. p values are indicated (chi-square test).

**(B)** Different levels of P-gp expression was relative to the progression of NMIBC. p values are indicated (chi-square test).

**(C)** Comparison of the RFS times of NMIBC patients with different levels of P-gp expression. p values are indicated (log-rank test).

**(D)** Comparison of the PFS times of NMIBC patients with different levels of P-gp expression. p values are indicated (log-rank test).

**(E)** Percentage of MIBC specimens with or without recurrence relative to the high or low P-gp expression. p values are indicated (chi-square test).

**(F)** Comparison of the OS times of MIBC patients with different levels of P-gp expression. p values are indicated (log-rank test).

**(G)** Comparison of the RFS times of MIBC patients with different levels of P-gp expression. p values are indicated (log-rank test).

**(H)** OS curves in MIBC patients in P-gp low group who underwent radical cystectomy with or without adjuvant chemotherapy. p values are indicated (log-rank test).

**(I)** OS curves in MIBC patients in P-gp high group who underwent radical cystectomy with or without adjuvant chemotherapy. p values are indicated (log-rank test).

**Supplementary Table 1. The baseline clinicopathologic features of 115 MIBC patients**

|  | Adjuvant Therapy  (n=57) | Non Adjuvant Therapy (n=58) | P value |
| --- | --- | --- | --- |
| Age, years |  |  |  |
| Median (IQR) | 61 (51-69) | 68 (59-72) | ***0.003*** |
| Gender |  |  |  |
| Male | 51 (89.5%) | 51 (87.9%) | 0.794 |
| Female | 6 (10.5%) | 7 (12.1%) |
| T stage |  |  |  |
| T2 | 5 (8.8%) | 6 (10.3%) | 0.779 |
| T3-4 | 52 (91.2%) | 52 (89.7%) |
| N stage |  |  |  |
| N0-1 | 42 (73.7%) | 40 (69.0%) | 0.919 |
| N2-3 | 15 (26.3%) | 18 (31.0%) |
| M stage |  |  |  |
| M0 | 53 (93.0) | 57 (98.3%) | 0.360 |
| M1 | 4 (7.0) | 1 (1.7%) |
| DFS, months |  |  |  |
| Median (IQR) | 27.0 (9.1-45.5) | 8.0 (3.8-33.3) | ***0.019*** |
| OS, months |  |  |  |
| Median (IQR) | 33.0 (15.2-52.0) | 16.0 (8.0-41.3) | ***0.037*** |

MIBC: muscle invasive bladder cancer; DFS: disease-free survival; OS: overall survival

**Supplementary Table 2. Correlation between the clinicopathologic features and expression of Nkx2.8 and P-gp in 131 NMIBC**

| Characteristics | Total  (n=131) | Nkx2.8 | *p* value | P-gp | *p* value |
| --- | --- | --- | --- | --- | --- |
|  |  | Negative Positive  54.96% 45.04% |  | Low High  32.06% 67.94% |  |
| Gender  Male  Female | 111  20 | 59(81.9) 52(88.1) 13(18.1) 7(11.9) | 0.327 | 39(92.9) 72(80.9)  3(7.1) 17(19.1) | 0.076 |
| Age(y)  ≥63  ＜63 | 68  63 | 35(48.6) 33(55.9) 37(51.4) 26(44.1) | 0.404 | 25(59.5) 43(48.3)  17(40.5) 46(51.7) | 0.231 |
| Grade  Low  High | 38  93 | 25(34.7) 13(22.1) 47(65.3) 46(77.9) | 0.111 | 13(30.9) 25(28.1)  29(69.1) 64(71.9) | 0.736 |
| Tumor size  ≥2.5cm  ＜2.5cm | 66  65 | 33(45.8) 33(55.9) 39(54.2) 26(44.1) | 0.250 | 21(50.0) 45(50.6)  21(50.0) 44(49.4) | 0.952 |
| Number  Single  Multiple | 53  78 | 29(40.3) 24(40.7) 43(59.7) 35(59.3) | 0.963 | 22(52.4) 31(34.8)  20(47.6) 58(65.2) | 0.056 |
| Recurrence  Yes  No | 73  58 | 50(69.4) 23(39.0) 22(30.6) 36(61.0) | ***0.0005*** | 14(33.3) 59(66.3)  28(66.7) 30(33.7) | ***0.0004*** |
| Progression  Yes  No | 46  85 | 35(48.6) 11(18.6) 37(51.4) 48(81.4) | ***0.0004*** | 5(11.9) 41(46.1)  37(88.1) 48(53.9) | ***<0.0001*** |

NMIBC: Non-muscle invasive bladder cancer

**Supplementary Table 3. Univariate and multivariate analysis of various prognostic parameters for recurrence free survival of 131 NMIBC patients**

|  | Univariate analysis | |  | | Multivariate analysis | | |  |
| --- | --- | --- | --- | --- | --- | --- | --- | --- |
|  | Hazard ratio (95% CI) | *p* value |  | | Hazard ratio (95% CI) | | *p* value |  |
| Nkx2.8 | 0.766 (0.637~0.903) | ***0.001*** | | 0.953 (0.766~1.166) | | 0.655 | | |
| P-gp | 1.180 (1.089~1.285) | ***<0.0001*** | | 1.120 (1.011~1.247) | | ***0.029*** | | |
| Tumor size | 1.306 (1.098~1.537) | ***0.003*** | | 1.414 (1.162~1.727) | | ***0.0006*** | | |
| Number | 1.088 (1.021~1.159) | ***0.0096*** | | 1.073 (1.0001~1.151) | | ***0.049*** | | |
| Grade | 1.937 (1.104~3.548) | ***0.020*** | | 1.856 (1.015~3.515) | | ***0.044*** | | |

NMIBC: Non-muscle invasive bladder cancer

**Supplementary Table 4. Univariate and multivariate analysis of various prognostic parameters for progression free survival of 131 NMIBC patients**

|  | Univariate analysis | |  | | Multivariate analysis | | |  |
| --- | --- | --- | --- | --- | --- | --- | --- | --- |
|  | Hazard ratio (95% CI) | *p* value |  | | Hazard ratio (95% CI) | | *p* value |  |
| Nkx2.8 | 0.745 (0.583~0.923) | ***0.005*** | | 0.018 (0.764~1.317) | | 0.896 | | |
| P-gp | 1.257 (1.138~1.340) | ***<0.0001*** | | 1.274 (1.110~1.475) | | ***0.0004*** | | |
| Tumor size | 1.393 (1.121~1.707) | ***0.003*** | | 1.633 (1.244~2.174) | | ***0.0004*** | | |

NMIBC: Non-muscle invasive bladder cancer

**Supplementary Table 5. Sequences of primers for real-time PCR (5' to 3')**

| Gene | Forward primer | Reverse primer |
| --- | --- | --- |
| Nkx 2.8 | CTGGACGCCTGAGCTTCAC | TAGCACCCGCCGCTTCTT |
| MDR1 | AATGCGACAGGAGATAGG | TGTTGCCATTGACTGAAA |
| GAPDH | GACTCATGACCACAGTCCATGC | AGAGGCAGGGATGATG TTCTG |

**Supplementary Table 6**. Sequences of primers for ChIP assays (5' to 3')

| *MDR1* ChIP primer | Forward primer | Reverse primer |
| --- | --- | --- |
| Primer 1 | GGCTTCACTGGAGTTATT | ACCTGGCATTGTCTTTAC |
| Primer 2 | GTCAGGGAGGTTTCACAT | GCCTTAACCCTAAAGAGG |
| Primer 3 | CCCTGCCCTCACTAAACT | ATGTATTGTGGGATCTGG |
| Primer 4 | CACAGATTCCAAAATGCA | GTGTTCCAAAGAAATGTT |
| Primer 5 | TAGCCAGTGGATAAAGAG | AACCTCGAAGAGTTACAT |
| Primer 6 | CTTCTCAACTCTGGCTAT | CAAGGATAAGTTTGGGTG |
| Primer 7 | TTAGTTCATGTAGCTCCTC | ACCTTACCTTTTATCTGG |
| Primer 8 | TCTTTGGTTTCATCTCAG | CACATTTATCGAATAATAGG |
| Primer 9 | AAGCCATGTTTCTGTTTG | AATGATTGGGTGGGGTAG |
| Primer 10 | TGAGCTACTCGATTCTGA | ACTTCTTTTCAAGCAACA |
| Primer 11 | TCTTTCTATCCTGGTCAA | CCTAATACAGGGTTTGAC |
| Primer 12 | CTCCGACCAATAGAACCT | TAATGTAGCCACTAACAC |
| Primer 13 | AGATCAGCAGATGAGTGC | TCTTGGATTGTATAAAGATG |
