# Supplementary figures and images for: Nkx2.8 promotes chemosensitivity in bladder urothelial carcinoma via transcriptional repression of MDR1
Source: Cell Death Dis. 2022 May 24;13(5):492. doi: 10.1038/s41419-022-04947-x (PMC9130207; doi:10.1038/s41419-022-04947-x)

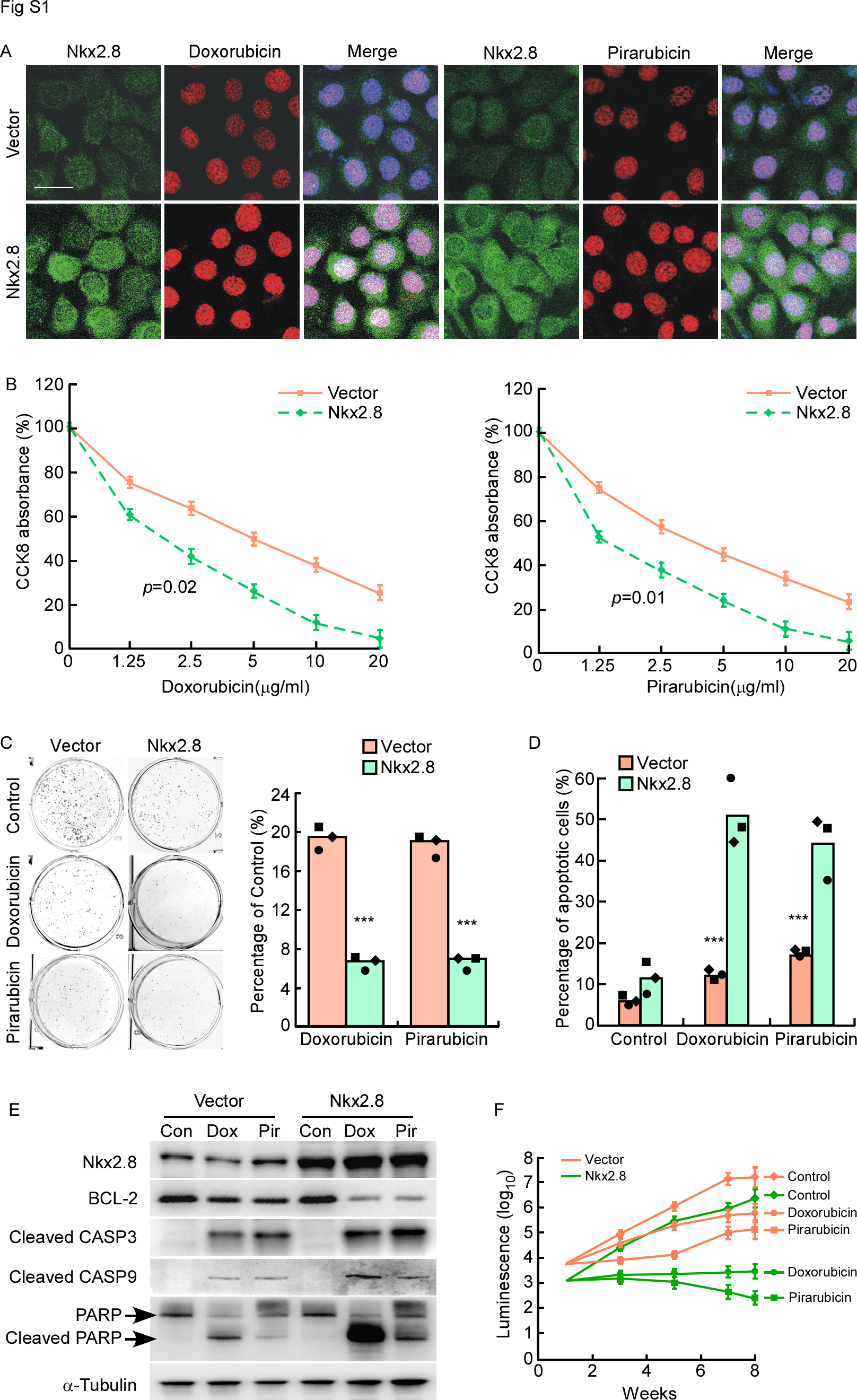

Supplement: Supplementary file 2 — Supplemental Figure 1 [file 41419_2022_4947_MOESM2_ESM.tif]

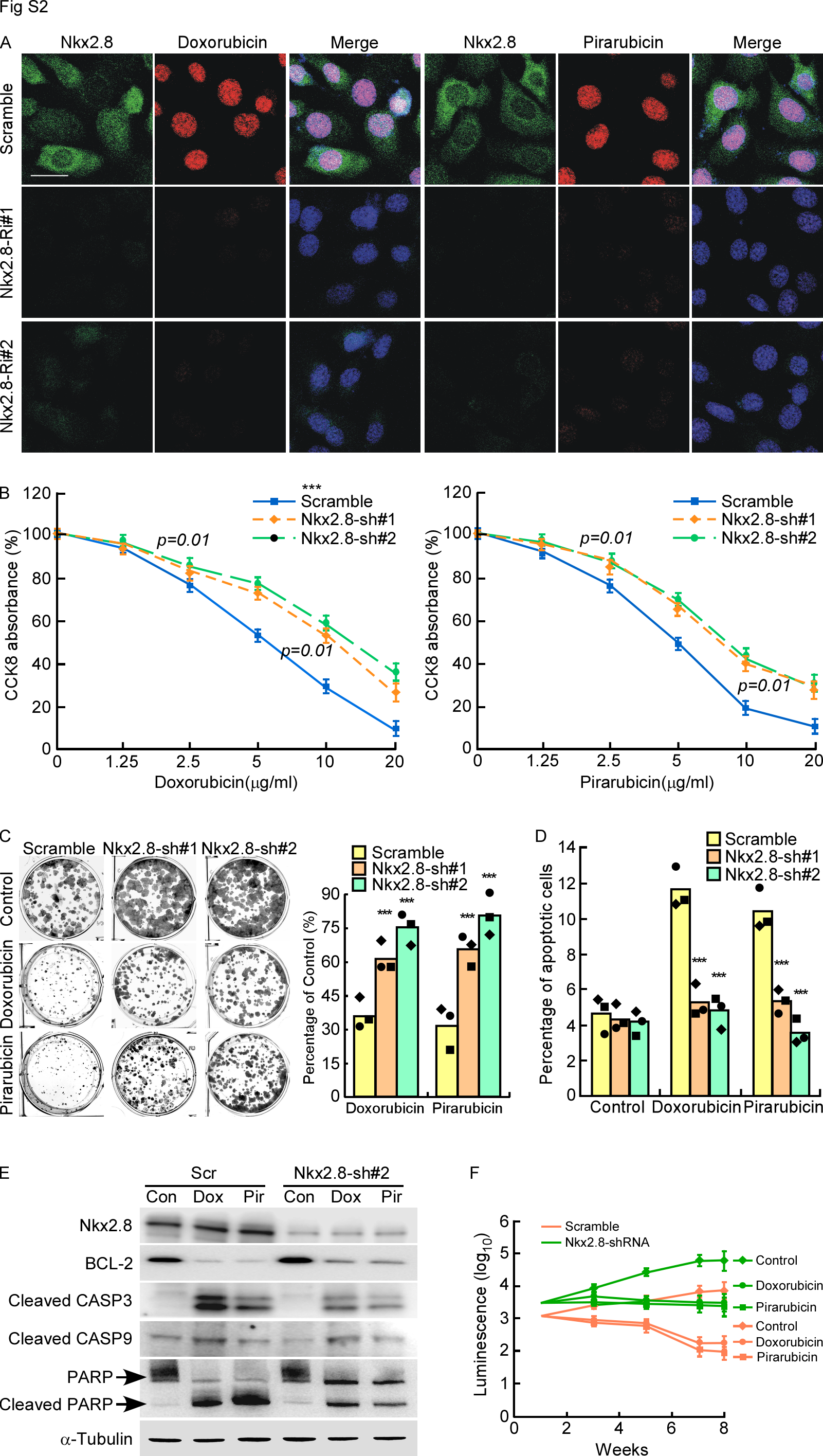

Supplement: Supplementary file 3 — Supplemental Figure 2 [file 41419_2022_4947_MOESM3_ESM.tif]

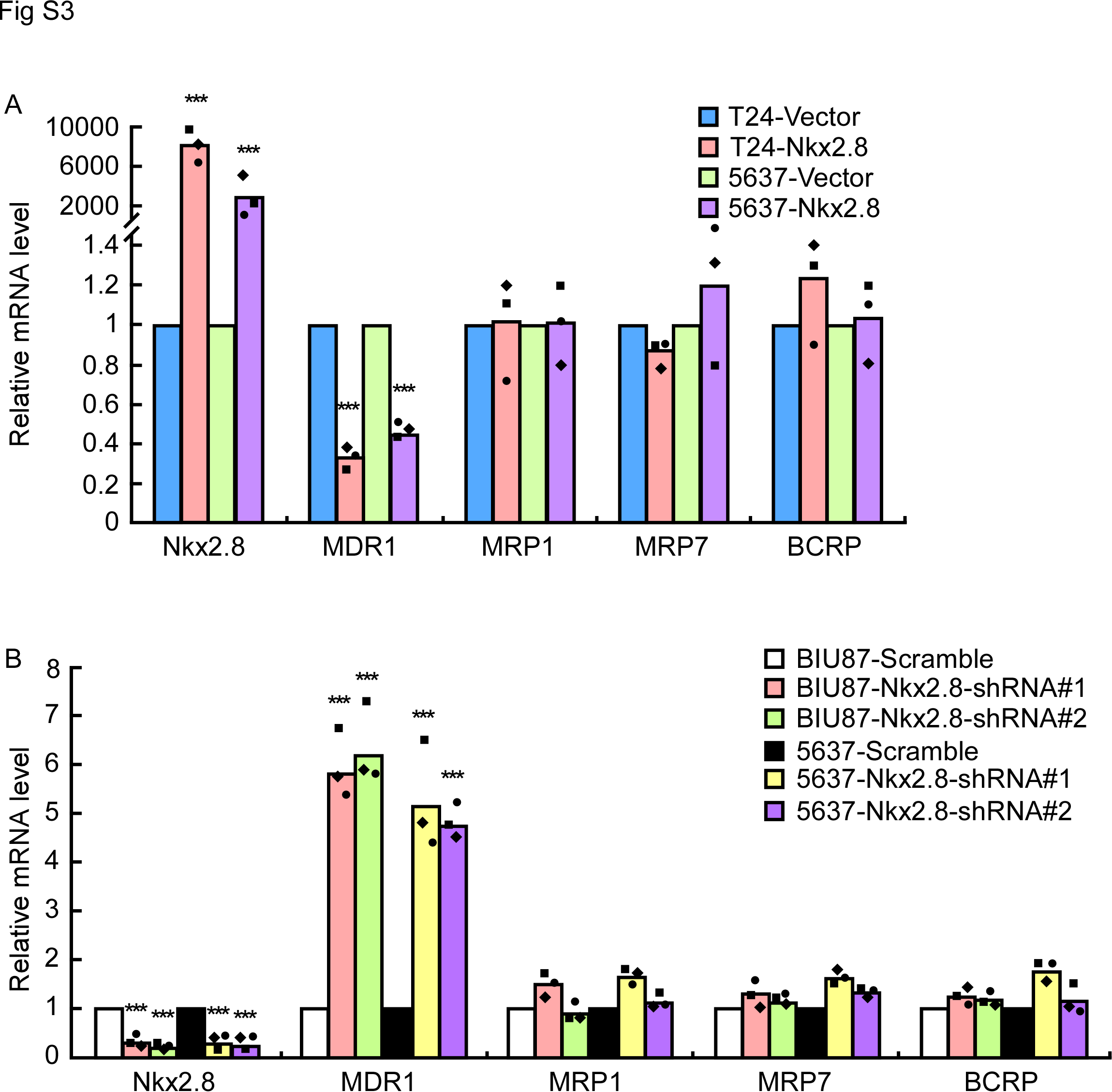

Supplement: Supplementary file 4 — Supplemental Figure 3 [file 41419_2022_4947_MOESM4_ESM.tif]

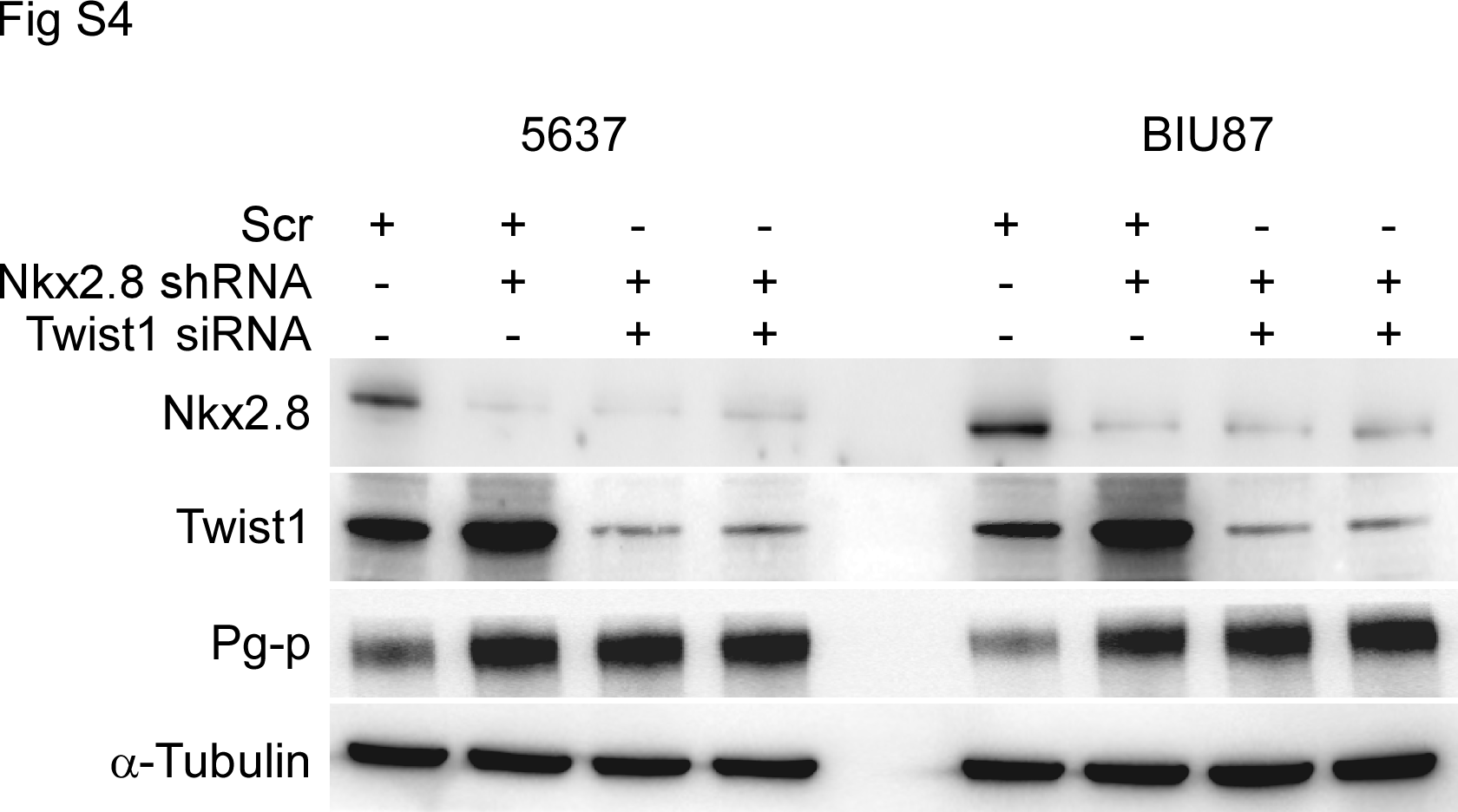

Supplement: Supplementary file 5 — Supplemental Figure 4 [file 41419_2022_4947_MOESM5_ESM.tif]

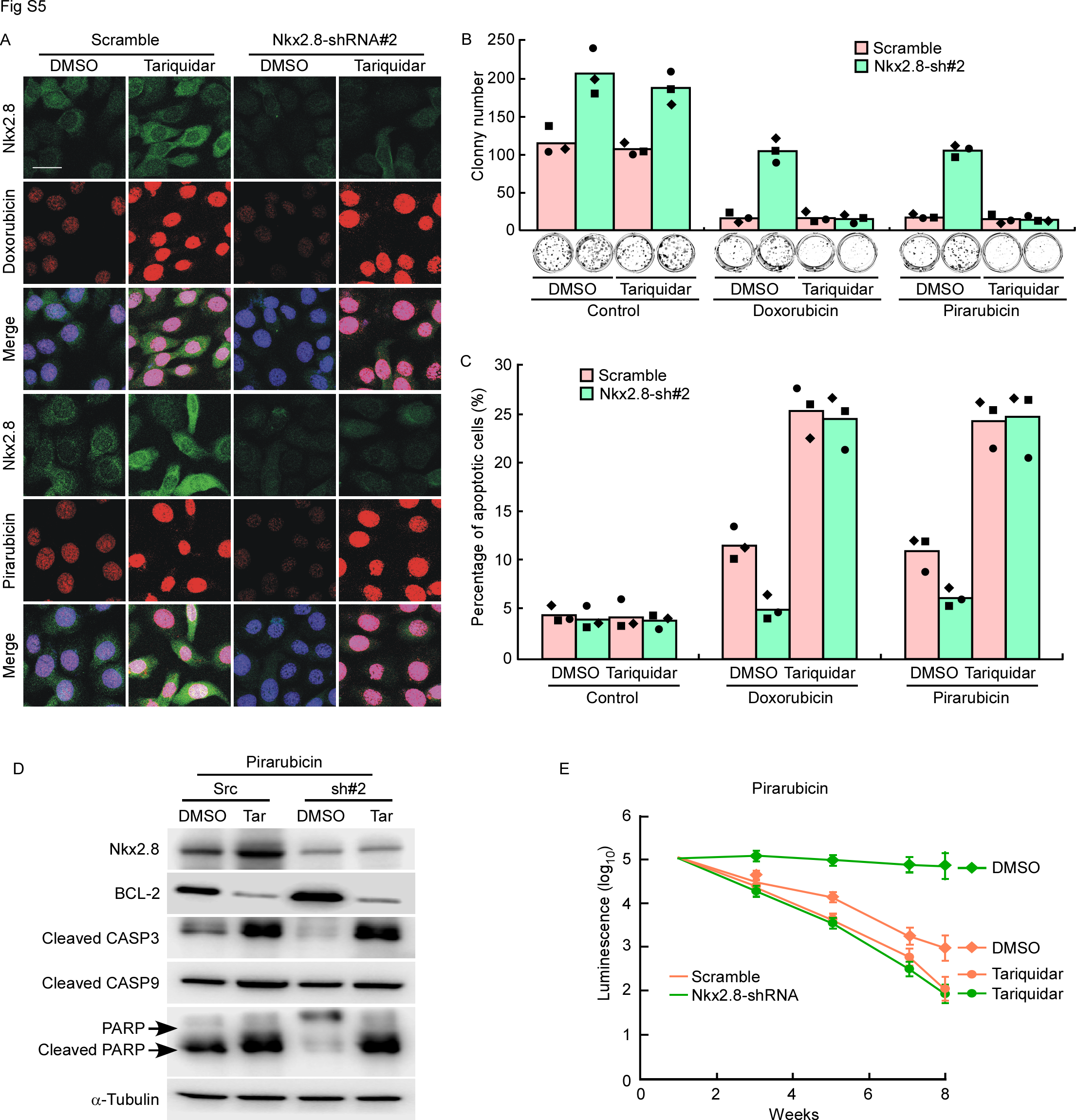

Supplement: Supplementary file 6 — Supplemental Figure 5 [file 41419_2022_4947_MOESM6_ESM.tif]

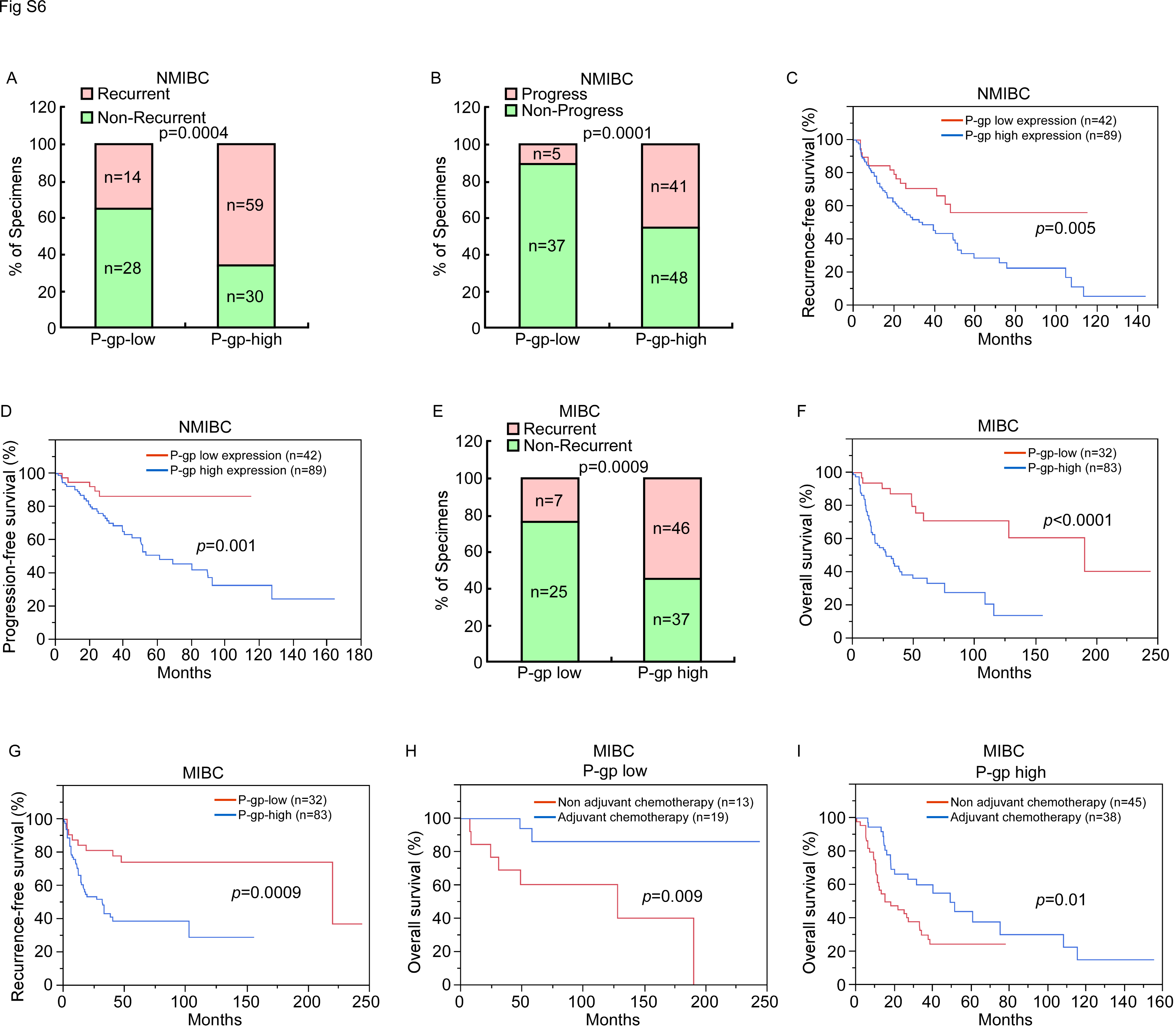

Supplement: Supplementary file 7 — Supplemental Figure 6 [file 41419_2022_4947_MOESM7_ESM.tif]

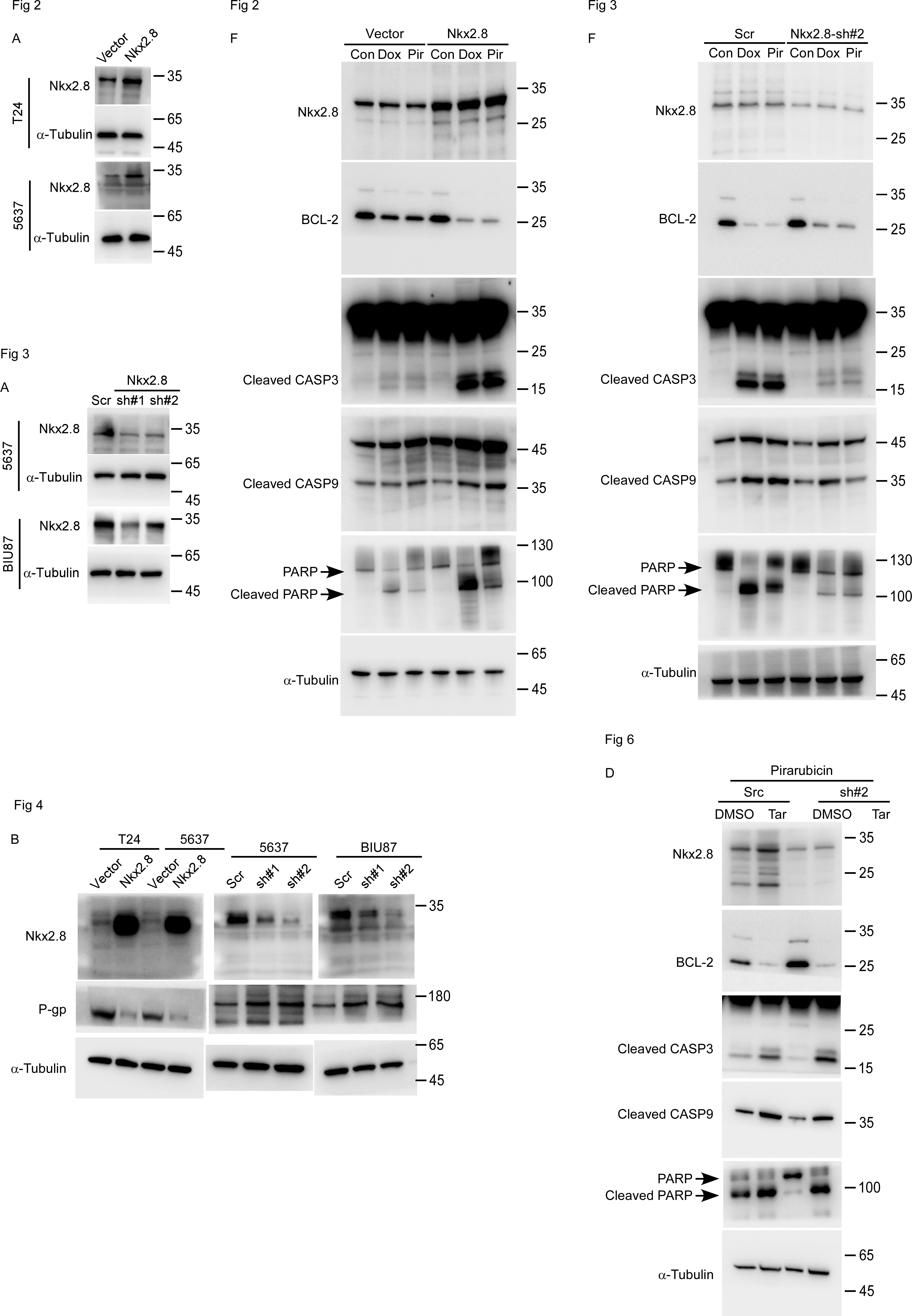

Supplement: Supplementary file 8 — Original Data File [file 41419_2022_4947_MOESM8_ESM.tif]
